# Low lean mass and chemotherapy toxicity risk in the elderly: the Fraction study protocol

**DOI:** 10.1186/s12885-019-6377-7

**Published:** 2019-11-27

**Authors:** Zara Steinmeyer, Stéphane Gérard, Thomas Filleron, Stéphanie Lozano, Delphine Brechemier, Gabor Abellan Van Kan, Loic Mourey, Laurence Cristol-Dalstein, Laure De Decker, Yves Rolland, Laurent Balardy

**Affiliations:** 1 Geriatric Department, Internal Medicine and Oncogeriatry Unit, University Hospital, Place du Docteur Baylac, TSA 40031, 31059 Toulouse Cedex 9, France; 2 Department of Biostatistics, Institut Claudius Regaud, IU, CT-O, Toulouse, France; 3 Medical oncology department, Claudius Régaud Institute-Oncopole-Toulouse Cancer University Institute (IUCT-O), 1 avenue Irène Joliot-Curie, 31100 Toulouse, France; 4 Oncogeriatry, ICM Val d’Aurelle, Montpellier, France; 5 Clinical Gerontology Department, Centre Hospitalier Universitaire de Nantes, F-44000 Nantes, France; 6 EE MiHAR (Microbiotes, Hôtes, Antibiotiques et Résistance bacterienne), Institut de Recherche en Santé (IRS2), Université de Nantes, F-44200 Nantes, France; 7 Gerontopole of Toulouse, Institute of Ageing, Toulouse University Hospital (CHU Toulouse), Toulouse, France; 8 UPS/Inserm UMR1027, University of Toulouse III, Toulouse, France; 9 UMR, INSERM, 1027 University of Toulouse III, Toulouse, France

**Keywords:** Aged, Chemotherapy toxicity, Low lean mass, Appendicular lean mass, Muscle mass, Dual energy X-ray absorptiometry, Cancer

## Abstract

**Background:**

Half of cancer cases occur in patients aged 70 and above. Majority of older patients are eligible for chemotherapy but evidence for treating this population is sparse and severe toxicities affect more than half of them. Determining prognostic biomarkers able to predict poor chemotherapy tolerance remains one of the major issues in geriatric oncology. Ageing is associated with body composition changes (increase of fat mass and loss of lean mass) independently of weight-loss. Previous studies suggest that body composition parameters (particularly muscle mass) may predict poor chemotherapy tolerance. However, studies specifically including older adults on this subject remain sparse and the majority of them study body composition based on computed tomography (CT) scanner (axial L3 section) muscle mass estimation. This method is to date not validated in elderly cancer patients.

**Methods:**

This trial (Fraction) will evaluate the discriminative ability of appendicular lean mass measured by dual-energy X-ray absorptiometry (DXA) to predict severe toxicity incidence in older cancer-patients treated with first-line chemotherapy. DXA is considered the gold standard in body composition assessment in older adults.

Patient’s aged ≥70 diagnosed with solid neoplasms or lymphomas at a locally advanced or metastatic stage treated for first-line chemotherapy were recruited. Patients completed a pre-chemotherapy assessment that recorded socio-demographics, tumor/treatment variables, laboratory test results, geriatric assessment variables (function, comorbidity, cognition, social support and nutritional status), oncological risk scores and body composition with DXA. Appendicular lean mass was standardized using evidence based international criteria. Participants underwent short follow-up geriatric assessments within the first 3 months, 6 months and a year after inclusion. Grade 3 to 5 chemotherapy-related toxicities, as defined by the National Cancer Institute Common Terminology Criteria for Adverse Events (NCI CTCAE) were assessed at each chemotherapy cycle.

**Discussion:**

The finding that body composition is associated with poor tolerance of chemotherapy could lead to consider these parameters as well as improve current decision-making algorithms when treating older adults.

**Trial registration:**

ClinicalTrials.gov Identifier: NCT02806154 registered on October 2016.

## Background

The risk of developing cancer increases with age [1]. Majority of older patients are eligible for chemotherapy but evidence for treating this population is sparse often due to their underrepresentation in clinical trials [2, 3]. Approximatively half of the older patients will present severe toxicity (defined as a toxicity above grade 3 by the common toxicity criteria (NCI-CTC Version 3.0) [2, 4], suggesting that modalities of chemotherapy in older adults should be adapted.

The elderly patient population is very heterogeneous because of the presence of various geriatric syndromes (dementia, urinary incontinence, loss of autonomy, falls, undernutrition …), number and severity of co-morbidities and level of cognitive and physical performances. This heterogeneity induces a large variability in chemotherapy tolerance. Predicting tolerance in older adults treated with chemotherapy can be a complicated task, leading to potential inappropriate care plan. Finding predictive factors of chemotoxicity, functional decline, poor quality of life, or early death has become a growing topic of research in geriatric oncology.

The Comprehensive Geriatric Assessments (CGA) approach is currently recommended in older adults by the international society of geriatric oncology to predict toxicity risk in addition to oncological parameters [3, 5]. However, performance of a CGA in predicting chemotherapy toxicity has been reported to be low [6]. New clinical tools integrating larger clinical geriatric parameters such as the Chemotherapy Risk Assessment Scale for High-Age Patients (CRASH) [7] or the Cancer and Aging Research Group (CARG) [8], have been developed but specific inter-individual variations remain difficult to capture, suggesting that other parameters that weigh on prognosis are currently not taken into account.

Evidence supports that body composition may be an important predictor of chemotherapy toxicity in older adults with cancer [9, 10]. Body Mass Index (BMI) is a basic proxy of body composition (mainly fat mass) and is recognized as an independent risk factor of adverse outcomes of chemotherapy [11, 12].

However, BMI does not capture the age-related changes in body composition such as the increase in fat tissue and a decrease in lean mass in particular muscle mass that can occur independently of weight-loss [13]. We recently reported that the relationship between body composition and in particular muscle mass with chemotherapy tolerance has been repeatedly found in adults but never specifically with older patients [9]. Yet, the aged-associated changes in body composition are variable from one individual to another but can be very important and significantly affect drug pharmacokinetics. Our hypothesis is that body composition in older cancer patients may, at least in part, explain heterogeneity of chemotherapy tolerance between older cancer patients [9, 10, 14, 15].

The objective of this manuscript is to report the study design of the Fraction study, an ongoing prospective multicenter cohort aiming to evaluate whether Appendicular Lean Mass (ALM) predicts the incidence of severe chemo-toxicity in older cancer patients treated with chemotherapy.

## Research hypothesis

Our hypothesis is that body composition of older cancer patients and especially ALM, is a significant factor affecting drug pharmacokinetics and is associated with chemotherapy tolerance in older cancer patients. In the future, a better understanding of the relationship between body composition and chemotherapy tolerance in older adults may contribute to better decision-making algorithms and perspectives of chemotherapy doses adjusted on body composition.

## Methods/design

### Objectives of the study

The principal objective is to evaluate the ability of ALM (measured by dual-energy X-ray absorptiometry (DXA)) to predict the incidence of severe toxicity grade 4 hematologic or grade 3 to 4 non hematologic toxicity (as defined by the National Cancer Institute-Common Toxicity Criteria (NCI-CTC version 3)) [16] and/or chemotherapy interruption related to unacceptable toxicity in older cancer-patients treated with first-line chemotherapy for solid neoplasm or lymphoma.

The following are the secondary objectives:

To evaluate various other body composition parameters: total lean mass, total fat mass, ALM standardized by squared height or body mass index (ALM / height^2^, ALM / BMI), an index of ALM / appendicular fat mass, total lean mass index (total lean mass/ height^2^), total fat mass index (total fat mass / height^2^) in predicting toxicity. These parameters, will then be studied to assess their ability to predict functional decline (defined as a loss of ≥0.5 points in ADL score [17] during follow-up), physical performance decline (defined as a loss ≥1 point in the SPPB [18] during follow-up), quality of life decline (defined as a loss ≥10 points in the EORTC QLQ-C30 questionnaire [19] during follow-up) and early death (defined as death within the 3 first months of the first-line of chemotherapy).

Moreover, a toxicity risk profile will be studied by using combined scores including parameters of body composition and a geriatric assessment. This toxicity profile will be compared to current existing toxicity scores such as the CRASH score [7], CARG [8] and the G8 [20].

### Study design

The Fraction study is an ongoing prospective multicenter cohort, coordinated by the CHU of Toulouse, (https://clinicaltrials.gov/ct2/show/NCT02806154) which evaluates the discriminant ability of ALM measured by DXA to predict the incidence of severe chemo-toxicity in older adults with cancer treated with first line chemotherapy for solid neoplasm or lymphoma.

### Participants

Eligible patients are above 70 years of age and are diagnosed with solid neoplasms (breast, prostate, bladder, colo-rectal, ovarian cancers) or lymphomas at a locally advanced or metastatic stage treated for first-line chemotherapy with a life expectancy above 3 months.

Patients treated by a combination of chemotherapy with targeted therapies or radiotherapy were excluded due to the potential difficulty to measure the isolated effects of chemotherapy.

Patients whose weight exceeds 136 kg and height is above 196 cm are excluded because it is the limit for the DXA table and may induce errors in assessing body composition, injury to the patient and damage to the DXA table.

Other hemopathies than lymphoma were excluded because their potential treatments vary and they have various toxicity responses.

Cognitive status was evaluated with Mini-Mental Status Examination (MMSE) and the cut off score of 20 was chosen to exclude certain participants due to difficulty of follow-up, providing informed consent and safety concerns. Moreover, patients under protection measures are excluded for the same reasons.

Inclusion and exclusion criteria are summarized in Table 1.

**Table 1:** Fraction study inclusion and exclusion criteria

Inclusion criteria: 1. Age ≥ 70 years old 2. Cancer types: Breast, prostate, bladder, colo-rectal, ovarian cancers, and lymphoma - Metastatic or locally advanced neoplasm 3. First-line chemotherapy 4. Performance status World Health Organization (WHO) score 0 to 3 5. Capacity to give a written informed consent 6. Life expectancy > 3 months Exclusion criteria: 1. Targeted therapies in combination with chemotherapy 2. Radiotherapy in combination with chemotherapy 3. Height > 196 cm and weight > 136 kg (DXA not feasible) 4. Hemopathy excluding lymphoma 5. Cognitive impairment (MMSE < 20/30) due to difficulty of follow-up and providing informed consent	

Recruitment will be performed by four geriatric oncology units: Midi-Pyrénées; Provence Alpes Côte d’Azur, Pays de la Loire and Languedoc Roussillon (Toulouse University hospital, Claudius Regaud institute of Toulouse, Nice University hospital, Nantes University hospital and the Val Aurel institute of Montpellier).

Participants will be recruited over 3 years, beginning in March 2017. The study goal is to recruit 160 participants over a two-year period.

A team comprising an oncologist and geriatrician was chosen in each recruitment center to verify protocol compliance. Patients will be referred to these units by their oncologist, hematologist, onco-geriatrician or geriatrician. The study protocol was reviewed and approved by the Committee for the protection of persons of the southwest and overseas region III for all sites. All patients will sign the informed consent form prior to the study.

A screening visit will be conducted within a month before the baseline visit by a study coordinator to verify eligibility criteria and protocol aspects will be discussed with the patient.

Once the patient’s informed consent has been obtained a baseline visit will be scheduled.

### Data collection

Patient related information age, gender, socio-economic status and education, social support and medication are recorded.

#### DXA parameters

Measurement of body composition parameters by dual energy X-ray absorptiometry DXA, LUNAR iDXA GE, and HOLOGIC was performed by qualified technicians and cross calibration was done in between centers.

Indeed, the DXA machine was used to target a specific region of interest; appendicular lean mass, and underwent further calibration procedures due to the fact that two systems were used Hologic system© and the Lunar system© (iDXA, General Electrics). To limit result variability due to the use of different DXA machines and softwares, calibration was done using the same phantom following a standardized procedure.

Total and regional distribution of lean mass, bone mass and fat mass was estimated. Appendicular lean mass and fat mass were standardized using evidence-based international criteria.

#### Cancer characteristics and previous treatment

Tumor-specific variables included WHO performance status, primary tumor location (histologic grade, TNM classification …) and proposed cancer treatment strategies are recorded.

#### Geriatric assessment

The patient evaluation assisted by a health care team member consisted of measures of comorbidity using the score cumulative illness rating scale geriatric (CIRS-G) [21], psychological state with the geriatric depression scale (GDS 15) [22], functional autonomy Katz’s Activities of daily living (ADL) [23], Lawton’s instrumental activities of daily living (IADL) [17] and quality of life assessed by EORTC QLQC 30 [19]. The nutritional status was evaluated by loss of weight as a percentage, Mini Nutritional Assessment (MNA) [24] and Body Mass Index (BMI) defined by weigh devised by height square. Physical performance was assessed using the Short Physical Performance Battery SPPB [18] a composite score of chair stand, walking speed and balance test.

The physicians then performed a clinical examination, a cognitive assessment with the Mini Mental State Examination (MMSE) [25] and completed series of oncological risk scores: the G8 score [20], the cancer and aging research group CARG score [8] and the chemotherapy risk assessment scale for high CRASH [7].

Pretreatment laboratory data including complete blood counts (white blood cell, neutrophil, lymphocyte, platelet values), albumin, creatinine, C-reactive Protein (CRP) and lactate dehydrogenase (LDH) were recorded before first-line chemotherapy was administered.

#### Chemotherapy toxicity

Chemotherapy toxicity (hematological and non-hematological toxicity) is assessed at each chemotherapy cycle during scheduled or emergency visits with the patient’s oncologist according to their cancer plan within the one-year follow-up according to the national cancer institute common terminology criteria for adverse events (NCI CTCAE), version 3.0 [16] based on questioning, physical examination, and laboratory tests. Toxicity is graded as mild (Grade 1), moderate (Grade 2), severe (Grade 3), life-threatening (Grade 4) or fatal (Grade 5). The principal investigator and site sub-investigator will review patient’s chemotherapy course to confirm any Grade 3 to 5 declared chemotherapy-related toxicity.

In order to compare different chemotherapy protocols, the MAX 2 index will be used to estimate the average per patient risk for chemotherapy toxicity. This procedure has been previously used in research [7].

Treatment benefits is regularly assessed by the oncologist and is classified using the Response Evaluation Criteria in Solid Tumors (RECIST 1.1) [26] for solid neoplasms, or by the revised Cheson criteria for lymphomas [27]. Treatment will be interrupted in case of disease progression or unacceptable toxicity as per the oncologist’s discretion.

### Study scheme

Baseline visit (including DXA assessment) is organized in a day care hospital unit where a geriatrician and a nurse will conduct the previous extensive comprehensive geriatric assessment.

Patient’s follow-up will be pursued according to their cancer care plan by their oncologist. Visit frequency depends on the cancer type and the treatment plan. The oncologist will perform a clinical examination and record chemotherapy toxicity according to the WHO toxicity grading system at each visit [16].

**Fig. 1:**
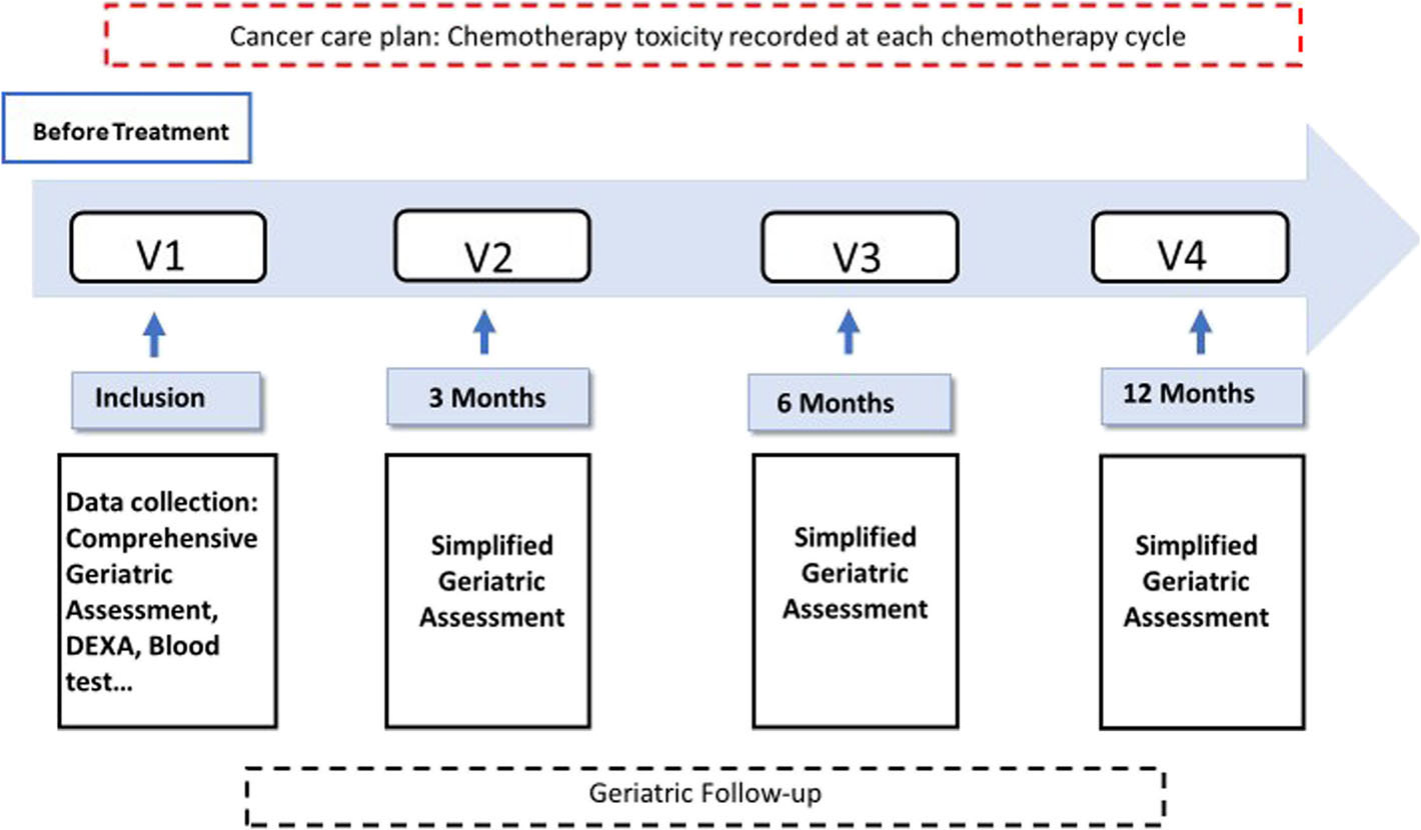
Assessment and follow up study scheme

Participants are asked to return for simplified follow-up geriatric assessments within the first 3 months, 6 months and a year after inclusion. At this visit, a nurse trained in onco-geriatrics will perform during the visit measures of functional status (ADL, IADL) [17], nutrition (weight, MNA [28], BMI), physical performance (SPPB) [18] and the European organization for research and treatment of cancer quality of life score (QLQC-30) [19]. (Fig. 1).

Survival data will be collected for all patients during the study period.

### Statistical analysis

#### Sample size calculation and primary analysis

The primary objective of our study is to evaluate the discriminant ability of ALM to predict severe toxicity or chemotherapy interruption due to unacceptable toxicity. Discriminant ability will be evaluated by calculating the area under the receiver operation characteristic (ROC) curve (AUC). Incidence above grade > 2 toxicity occurs in about 50% of the elderly treated with chemotherapy [8]. Supposing a toxicity rate of 50%, 160 patients were required to estimate a 95% confidence interval of an AUC equal to 75% with an accuracy of +/− 7.5% [29].

#### Secondary endpoint analysis

Secondary objectives, which correspond to the evaluation of discriminant ability, will be evaluated using similar methodology as used in the primary endpoint.

To determine a toxicity model associated with the occurrence of severe toxicity, the population will be partitioned into two cohorts, a training cohort (first 100 patients recruited) and a validation cohort (last 60 patients recruited). This allows a temporal validation which can be considered an intermediate between internal and external validation. In the training cohort, a penalized logistic regression model with elastic net regularization [30] via the lasso will be used to identify associations between severe toxicities and different parameters (body composition, geriatric assessment parameters …) [31]. A 10-fold cross validation will be performed to select the best penalty lambda parameter. The other parameter of the Elastic net method, the mixing parameter α, will be set to a default value of 0.5 [32]. Using a resampling approach, bootstrap selection stability (BSS) will be computed for each parameter. Only parameters with high BSS will be selected in the final model. Based on the linear predictor given by the model, a classifier predicting severe toxicity will be computed. The risk group will be defined by dichotomizing the toxicity profile using the area under the ROC curve and will be estimated with corresponding 95% confidence interval (Hanley method) [33]. Risk group will be then applied on the validation cohort. Sensitivity and specificity of the toxicity risk group will be evaluated and compared to other published tools (CRASH [7], CARG [8], G8 [20]). All statistical analyses will be performed by using Stata v13.0 or R software.

### Data management and monitoring study

All findings including clinical, DXA and laboratory data will be documented by the investigator or an authorized member of the study team in the subject’s medical record and in the eCRF (Software Clinsight® edited by Ennov Society) managed by the Data Management cell (DMC Claudius Regaud institute). This DMC is an independent organization and has no competing interests. Data will be stored and encrypted for 15 years by an online secure site (https://ec.claudiusregaud.fr/CSOnline) accessible only with individual username and password. All data entry, modification or deletion will be recorded automatically in an electronic audit trail. Investigators are responsible for ensuring that all sections of the eCRF are completed correctly and that entries can be verified against source data Investigators guarantee the privacy of patients and personal data are treated according to French laws (article L.1121–3 and R.5121–13 from the French Public health code).

Investigators are also responsible for collecting adverse events following best clinical practice. The study procedure of FRACTION leads us to think that no serious adverse events will be recorded.

## Discussion

### Relevance of studying body composition in older patients

Low muscle mass is a major predictor of chemotoxicity in an adult cancer population [9]. Due to the cumulative effects of age, an older population is probably more concerned with the decline in muscle mass than the adult population but we do not have, to our knowledge, data attesting the impact of ALM on chemotherapy toxicity in elderly subjects. We believe that our work will improve reflexion to adjust chemotherapy protocols that are often complicated to do especially in the elderly taking into account their body composition. Gerard et al. in a systematic review studied the association of body composition in the elderly and chemotherapy tolerance and hypothesized that low muscle mass is associated with chemotherapy dose concession or severe toxicity (grade 3–4 NCICTC). A total of 24 studies were analyzed concerning different cancer localizations at different stages: colo-rectal, oesophagus, ovarian, breast cancers and non-Hodgkin lymphomas with different chemotherapy protocols either monotherapy or combined treatments (anthracyclines, taxanes, 5-Fluorouracil, cyclophosphamide) [9]. The mean age at inclusion was relatively young (under 60 years old) and the different study populations were small (maximum 93 patients) not allowing conclusions to be drawn on elderly subjects. To the best of our knowledge, no specific studies on body composition and chemotherapy tolerance in elderly cancer patients have yet been conducted.

What might explain the link between body composition and chemotoxicity? First, body composition compartments determine pharmacokinetics by influencing drug distribution according to the drugs liposolubility or to their protein binding ability [34].

Secondly, because the evaluation of body composition parameters is crucial in older adults due to their modification with age. Indeed, the loss of muscle mass and muscle strength (so-called sarcopenia) which is involved in the frailty concept predicts negative health outcomes such as risk of falls, loss of independence, institutionalization and death. Thus, the frail elderly have a higher chemotoxicity risk due to sarcopenia. Moreover, loss of muscle mass is one of the main determinants of cachexia, which is a huge pejorative prognostic factor in oncology.

### DXA to measure body composition

In a majority of cancer studies, body composition analyses are based on the estimation of muscle mass by a CT scanner due to their routine use in cancer work-ups and accessibility.

Total muscle mass is evaluated by quantitative measurements of muscle area from a single slice or muscle volume from a stack of slices covering a whole muscle. Horizontal images extending from the third lumbar vertebrae in the inferior direction were assessed for total muscle mass (psoas, paraspinal muscles and abdominal wall muscles) on a horizontal CT scan section [35]. These equations were highly correlated with DXA estimation. However, this method is not yet validated in elderly cancer patients. Robert D et al. have shown discordances between results from the Mourtzakis equation and DXA measurement. Concluding that these predictive methods aren’t reliable and cannot replace DXA use for the moment [36].

To date, DXA is considered as the gold standard in body composition assessment in older adults. This method is very precise and the weight calculated by the addition of non-fat mass and fat mass is approximatively at 1–2% precise [33–35].

Moreover, patient’s hydration status does not affect the equation except if the status exceeds 5% of the total body weight leading to an overstatement of the muscle mass [37, 38]. This method radiates poorly 0.037 mSv, DXA exposes less than 1300 mSv times of the maximal dose per year (20 mSv/0,111 mSv = 180). In the future, if body composition assessed is clinically relevant, the low degree of irradiation caused by DXA will allow for the safe repeating of measurements for patients who are already at high risk of radiation during CT scans.

### Choice of cancer types

Cancer types were selected prior to their frequency in the elderly (breast, prostate, colo-rectal, ovarian, bladder) and non-Hodgkin lymphomas. These 6 types of cancers represent over 60% of all women’s cancers (breast 44.4%; colon-rectum 12.2%; uterus 5,2%; ovarian 3.2%;lymphomas 2.8%) and 57% of all men cancers (prostate 36.6%; colon-rectum 13.3%; bladder 5.2%; lymphomas 2.7%) in patients aged above 65 years old in France [39].

### Study relevance

Identifying body composition parameters and in particular appendicular lean mass as a predictive factor of chemotherapy tolerance may have many implications:

- Improve predictive models of chemotoxicity by integrating these parameters to validated models (CARG, CRASH, comprehensive geriatric assessment (CGA));
- Improve treatment decision making;
- Routine use of DXA in pre-chemotherapy work-ups and improvement of correlation with CT scan.

Assessing the ALM could have associated benefits. Currently, chemotherapy is often adjusted taking into account renal function. However, low blood levels of creatinine can be explained by low muscle mass. In practice, an elderly person with low muscle mass will have an overestimation of his renal function and a high dosage of his chemotherapy even though his low muscle mass exposes him to greater chemotoxicity. Bretagne M et al. proves that using creatinine to estimate glomerular filtration rate of patients with low muscle mass leads to an increase of capecitabine toxicity due to overestimated renal filtration [40]. So changes in body composition should be considered when calculating chemotherapy doses, and taken into account when calculating the renal function.

Several authors have shown that chemotoxicity was correlated with drug dose per kilogram of lean mass [41]. However, the calculation of chemotherapy doses still remains in clinical practice based on the patient’s body surface area. This procedure is currently under examination by numerous experts [38–42].

Indeed, body surface area is a poor surrogate of patient’s variations of pharmacokinetics [43], toxicity risk [44] and it is not correlated with kidney or liver function [45]. As body composition and chemotherapy tolerance are closely linked, patient selection should be improved to enhance chemotherapy results. This could be particularly applicable in clinical practice and in therapeutic decision making, where an integrated approach could yield measurable results. Further, better patient selection would allow a more tailored treatment and provide a wider data set for patients under-represented in clinical trials. Better patient selection may also decrease impact of overtreatment and serious adverse events such as hospitalizations related to severe toxicity and disability.

Finally, this study will also assess the feasibility of DXA scanning in elderly cancer patients and could change our frailty geriatric approach in older cancer patients by adding a new predictive biomarker of negative health outcomes.

## Data Availability

Data sharing is not applicable to this article as no datasets were generated or analyzed during the current study.

## Abbreviations

ADL: Activities of daily living
ALM: Appendicular Lean Mass
AUC: Area under the curve
BMI: Body Mass Index
CARG: Cancer and Aging Research Group
CGA: Comprehensive Geriatric Assessment
CIRSG: Cumulative Illness Rating Scale Geriatric
CRASH: Chemotherapy Risk Assessment Scale for High-Age Patients
CRP: C-reactive Protein
CT: Computed Tomography
DXA: Dual-Energy X-ray Absorptiometry
EORTC QL-Q C30: The European Organization for Research and Treatment of Cancer Quality of Life Questionnaire
GDS: Geriatric depression scale
IADL: Instrumental Activities of Daily Living
LDH: Lactate Dehydrogenase
MMSE: Mini-Mental Status Examination
MNA: Mini Nutritional Assessment
NCI CTCAE: National Cancer Institute Common Terminology Criteria for Adverse Events
RECIST: Response Evaluation Criteria in Solid Tumors
ROC: Receiver operation characteristic
SPPB: Short Physical Performance Battery
WHO: World Health Organization
